# Adenoma receptors and histologic characteristics determining management outcomes

**DOI:** 10.1210/clinem/dgag180

**Published:** 2026-04-24

**Authors:** Luiz Eduardo Wildemberg, Mônica R Gadelha

**Affiliations:** Neuroendocrinology Research Center, Endocrinology Section, Medical School, and Hospital Universitário Clementino Fraga Filho, Universidade Federal do Rio de Janeiro, Rio de Janeiro 21941-913, Brazil; Neuroendocrine Unit, Instituto Estadual do Cérebro Paulo Niemeyer, Secretaria Estadual de Saúde, Rio de Janeiro 20231-092, Brazil; Neuroendocrinology Research Center, Endocrinology Section, Medical School, and Hospital Universitário Clementino Fraga Filho, Universidade Federal do Rio de Janeiro, Rio de Janeiro 21941-913, Brazil; Neuroendocrine Unit, Instituto Estadual do Cérebro Paulo Niemeyer, Secretaria Estadual de Saúde, Rio de Janeiro 20231-092, Brazil; Neuropathology and Molecular Genetics Laboratory, Instituto Estadual do Cérebro Paulo Niemeyer, Secretaria Estadual de Saúde, Rio de Janeiro 20231-092, Brazil

**Keywords:** acromegaly, somatostatin receptors, granulation pattern, precision medicine, biomarkers

## Abstract

Patient responses to medical therapy for acromegaly are variable. Adenoma features, including histologic characteristics and receptor density, are closely associated with distinct clinical behavior and therapeutic outcomes. Densely granulated pure somatotropinomas respond more favorably to somatostatin receptor subtype 2 (SST2)-selective ligands (octreotide and lanreotide) compared with sparsely granulated adenomas. In contrast, sparsely granulated adenomas may respond more favorably to pasireotide, which has a higher affinity for somatostatin receptor subtype 5 (SST5). Expression levels of SST2 receptors are also associated with responsiveness to octreotide and lanreotide, whereas response to pasireotide is linked to higher SST5 expression. Recent prospective studies have highlighted that treatment strategies guided by biomarkers determining therapeutic response are more effective than standard, non–biomarker-based approaches. Current evidence supports the incorporation of biomarkers into decision-making for medical management of acromegaly, while these approaches are being further validated.

Despite advances in surgical techniques, medical therapy, and radiotherapy, achieving disease control in acromegaly remains challenging in a significant subset of patients. Variability in treatment response is increasingly attributed to the underlying biological heterogeneity of somatotroph adenomas.

Histological somatotropinoma subtypes, such as sparsely and densely granulated pure adenomas, exhibit distinct clinical behaviors and therapeutic outcomes (1-3). Moreover, specific receptor profiles, particularly somatostatin receptors (SSTs), and other molecular targets have been shown to predict responsiveness to medical therapies (4, 5).

Understanding the interplay between histopathological features and receptor expression may enable tailored treatment strategies for acromegaly (6). This article reviews current evidence on adenoma histological characteristics and receptor expression profiles and evaluates how these factors influence management outcomes. Particular emphasis is placed on guiding personalized treatment, optimizing surgical and medical therapies, and improving long-term disease control in patients with acromegaly.

## Histological classification

According to the latest World Health Organization (WHO) classification, acromegaly originates from a PIT1-lineage adenoma, which can be classified as pure growth hormone (GH)-secreting adenoma; GH and prolactin co-secreting adenoma; mature or immature plurihormonal PIT1 adenoma; and very rarely, acidophil stem cell adenoma (7).

### Pure somatotropinomas

Pure somatotropinomas secrete GH exclusively, with no co-secretion of other anterior pituitary hormones, and they may be densely or sparsely granulated (7). The cytokeratin staining pattern is evaluated immunohistochemically using the CAM5.2 antibody, which detects low-molecular-weight cytokeratins (CK8/18) and serves as a reliable surrogate for ultrastructural analysis previously performed by electron microscopy (8). This histological classification correlates with key differences in magnetic resonance imaging (MRI) features, SST expression profiles, adenoma behavior, and response to both surgical and medical therapies (9-18).

Densely granulated somatotropinomas are composed of strongly acidophilic, densely granulated cells, showing diffuse and strong immunoreactivity for GH, closely resembling non-neoplastic somatotrophs histologically. They also express PIT1 and α-subunit and exhibit a diffuse or perinuclear cytokeratin staining pattern, reflecting the uniform distribution of intermediate filaments in these well-differentiated cells (7). In contrast, sparsely granulated somatotropinomas are composed of chromophobic cells with sparse secretory granules, showing only focal and weak immunoreactivity for GH (7). They exhibit diffuse PIT1 expression but lack α-subunit immunoreactivity and show a dot-like or fibrous body pattern, corresponding to intracytoplasmic aggregates of keratin filaments (7).

Clinical, biological, imaging, and pathological features of densely and sparsely granulated adenomas were compared in 131 patients (11). Patients with densely granulated adenomas had higher insulin-like growth factor 1 (IGF-I) levels before surgery, despite similar GH levels, and had smaller and less invasive sellar lesions that more frequently exhibited a hypointense signal on transverse relaxation time (T2)-weighted MRI. These adenomas were less proliferative and expressed higher somatostatin receptor subtype 2 (SST2) levels, both in terms of the percentage of positive cells and staining intensity. No significant difference in postsurgical remission was found between the 2 groups (11). On the other hand, it has been shown in a meta-analysis that patients with sparsely granulated adenomas had a lower chance of surgical remission (odds ratio [OR] 0.60; 95% confidence interval [CI]: 0.40-0.90; *P* = .01) (2). Although similar findings have been reported in other studies (14, 16, 19), most studies show similar surgical remission rates between patients with densely and sparsely granulated adenomas (11, 15, 17, 20, 21).

Adenoma granularity is well recognized as a predictor of response to treatment with somatostatin receptor ligands (SRLs). In a multicenter study in Brazil, including 153 patients with acromegaly naive to medical treatment and not in remission after surgery, densely granulated adenomas showed higher rates of biochemical control with octreotide or lanreotide when compared with sparsely granulated adenomas (46% vs 26%; *P* = .014) (22). A multicenter study in Italy found that patients with sparsely granulated adenomas had a 2.65-fold higher chance of not responding to octreotide or lanreotide treatment (23), similar to other studies (Table 1) (3, 10-18, 21-27).

**Table 1:** Correlation between granulation pattern and treatment response

Author, year	n	Multicenter study	Surgical outcome	Response to octreotide or lanreotide	Response to pasireotide
Bhayana, 2005 (12)	40	No	NA	SG showed lower biochemical response rate (IGF-I)	NA
Bakhtiar, 2010 (14)	141	Yes	SG showed lower surgical cure rate	SG showed lower GH reduction in AOT	NA
Fougner, 2012 (18)	78	No	NA	SG showed lower GH reduction in AOT and lower long-term IGF-I reduction	NA
Kato, 2012 (24)	82	No	NA	SG showed lower GH reduction in AOT	NA
Brzana, 2013 (17)	70	No	Similar cure rate	SG showed lower biochemical response rate (GH and IGF-I)	NA
Larkin, 2013 (3)	52	Yes	NA	SG showed lower GH reduction in AOT	NA
Sun, 2014 (21)	59	No	Similar cure rate	NA	NA
Iacovazzo, 2016 (25)	39	No	NA	Similar response rate (GH and IGF-I)	SG showed higher biochemical response rate (GH and IGF-I)
Antunes, 2018 (15)	69	No	Similar cure rate	NA	NA
Lasolle, 2019 (26)	15	No	NA	NA	SG showed higher biochemical response rate (IGF-I)
Dehghani 2021 (20)	71	No	Similar cure rate	Similar response rate (GH and IGF-I)	NA
Wildemberg, 2021 (10)	101	Yes	NA	SG showed lower biochemical response rate (GH and IGF-I)	NA
Wildemberg, 2021 (22)	153	Yes	NA	SG showed lower biochemical response rate (GH and IGF-I)	NA
Swanson, 2021 (11)	131	No	Similar cure rate	NA	NA
Tomasik, 2022 (19)	153	Yes	SG showed lower surgical cure rate	SG showed lower biochemical response rate (IGF-I)	Similar biochemical response rate (IGF-I)
Wang, 2024 (16)	40	No	SG showed lower surgical cure rate	NA	NA
Gliga, 2024 (27)	34	No	NA	SG showed lower biochemical response rate (IGF-I)	SG showed higher biochemical response rate (IGF-I)

Abbreviations: AOT, acute octreotide test; DG, densely granulated; GH, growth hormone; IGF-I, insulin-like growth factor 1; NA, not available; SG, sparsely granulated.

These findings were corroborated by a recent meta-analysis, which demonstrated that patients with sparsely granulated adenomas tend to be younger and present with larger, more invasive adenomas less responsive to octreotide and lanreotide, when compared with those with densely granulated adenomas (2).

In contrast, sparsely granulated adenomas have shown a more favorable response to pasireotide, an SRL that binds with the highest affinity to somatostatin receptor subtype 5 (SST5), followed by SST2, somatostatin receptor subtype 3 (SST3), and somatostatin receptor subtype 1 (SST1), compared with densely granulated adenomas (25-27).

### Co-secreting GH and prolactin adenomas

GH and prolactin (PRL) co-secreting adenomas can be either mammosomatotropinomas or mixed GH and PRL adenomas (7). The former resemble densely granulated somatotropinomas but additionally express PRL and estrogen receptor α within the same cells (7, 28). Mammosomatotropinomas are typically associated with florid acromegaly and PRL levels higher than those would be expected from a stalk effect alone (29). In contrast, mixed GH and PRL adenomas are composed of 2 distinct cell populations, each predominantly expressing 1 hormone (7, 28).

The frequency of co-secreting adenomas varies among studies, but they are estimated to occur in 21% to 30% of patients with acromegaly (29, 30). No sex difference has been observed; however, some reports suggest that mammosomatotroph adenomas may be more frequent in younger patients compared with mixed GH-PRL lesions (29). Mixed GH and PRL adenomas are usually larger, associated with PRL levels ≥200 ng/mL, and show lower surgical remission rates (31). It has also been shown that mixed GH and PRL adenomas present lower SST2 expression compared with mammosomatotroph adenomas, with similar SST5 expression (28).

### Comparisons between pure GH and co-secreting adenomas

In a study consisting of 604 patients (30), co-secreting adenomas, found in 21% of patients, were associated with a younger age at diagnosis, larger adenoma size, less frequent T2-weighted MRI hypointensity, lower IGF-I levels, and higher PRL levels (30). Rates of surgical remission and long-term biochemical remission with medical treatment were similar between pure GH and GH/PRL co-secreting adenomas. However, medical treatment was analyzed collectively rather than stratified by drug class (30).

In another study comparing co-secreting adenomas with pure sparsely and densely granulated somatotropinomas, co-secreting adenomas were smaller, with similar IGF-I levels (17). Surgical cure rates were comparable across the 3 groups; octreotide and lanreotide responses were similar to those observed for sparsely granulated adenomas and lower than for densely granulated tumors (17).

A multicenter British study found that IGF-I normalization was associated with GH/PRL co-secretion in 69 patients with acromegaly treated with cabergoline (32), and more patients with co-secreting tumors achieved IGF-I normalization when cabergoline was combined with octreotide or lanreotide (32).

On the other hand, it has been reported that GH/PRL adenomas required higher doses of cabergoline, lanreotide, and pegvisomant in 1 study (33).

Overall, it has been difficult to determine whether GH/PRL co-secretion predicts a more favorable response to SRLs or cabergoline (Table 2) (17, 19, 20, 28, 31-45).

**Table 2:** Correlation between adenoma subtypes and treatment response

Author, year	n	Surgery	Octreotide/lanreotide	Cabergoline	Pegvisomant
Colao, 1997 (34)	34	NA	NA	Co-secreting GH/PRL adenomas showed higher biochemical response rate	NA
Abs, 1998 (35)	64	NA	NA	Co-secreting GH/PRL adenomas showed higher IGF-I reduction than pure GH adenomas	NA
Cozzi, 2004 (36)	19	NA	NA	Similar biochemical remission with combination therapy	NA
Jallad, 2009 (37)	34	NA	NA	Similar biochemical remission with combination therapy	NA
Mattar, 2010 (38)	19	NA	NA	Similar biochemical remission with combination therapy	NA
Vilar, 2011 (39)	52	NA	NA	Similar biochemical remission with combination therapy	NA
Brzana, 2013 (17)	70	Similar cure rate	Co-secreting adenomas show biochemical response lower than DG and similar to SG	NA	NA
Suda, 2013 (40)	10	NA	NA	Similar biochemical remission with combination therapy	NA
Rick, 2018 (33)	91	Co-secreting GH/PRL adenomas show lower cure rate	Co-secreting GH/PRL adenomas require higher doses	Co-secreting GH/PRL adenomas require higher doses	Co-secreting GH/PRL adenomas require higher doses
Lv, 2019 (31)	94	Mixed GH/PRL adenomas show lower cure rate than pure GH and mammosomatotropinomas	NA	NA	NA
Sahin, 2020 (41)		NA	NA	Similar biochemical remission with combination therapy	NA
Dehghani, 2021 (20)	71	Similar cure rate	Similar biochemical remission rate	NA	NA
Kizilgul, 2022 (42)	50	NA	NA	Co-secreting GH/PRL adenomas show higher remission with combination therapy*^a^*	NA
Tomasik, 2022 (19)	153	Similar cure rate	Similar biochemical remission rate	NA	NA
Araujo-Castro, 2024 (30)	604	Similar cure rate	NA	NA	NA
Urwyler, 2024 (32)	69	NA	NA	GH/PRL adenomas show higher biochemical remission both for monotherapy and combination therapy	NA
Biagetti, 2024 (43)	144	NA	Co-secreting GH/PRL adenomas show lower biochemical remission rate	Similar biochemical remission with combination therapy	NA
Remya Rajan, 2025 (44)	30	NA	NA	Similar biochemical remission rate in monotherapy	NA
Chong, 2025 (45)	65	Similar cure rate	NA	NA	NA

Abbreviations: DG, densely granulated; GH, growth hormone; IGF-I, insulin-like growth factor 1; NA, not available; PRL, prolactin; SG, sparsely granulated.

^*a*^Borderline significance.

A validated classification distinguishing pure GH-secreting somatotroph adenomas from co-secreting GH/PRL subtypes is still lacking. PRL immunostaining alone is not sufficient to define an adenoma as co-secreting, because PRL is expressed in up to 5% of cells, likely reflecting intra-adenomatous entrapped normal adenohypophyseal cells rather than true hormonal co-secretion (46). This limitation leads to diagnostic variability, with inconsistent data on the true prevalence and biological behavior of these subtypes, and hinders meaningful comparisons across studies (47).

### Other PIT1-lineage adenomas associated with acromegaly

Other, less common subtypes that may be associated with acromegaly include mature and immature PIT1-lineage adenomas and acidophilic stem cell adenomas (7). Mature PIT1-lineage adenomas resemble mammosomatotroph adenomas but may exhibit variable expression of GH, PRL, or thyroid-stimulating hormone (TSH). In contrast, immature PIT1-lineage adenomas and acidophilic stem cell adenomas are composed of poorly differentiated cells that lack resemblance to terminally differentiated anterior pituitary cell types (7) and typically display more aggressive behavior and unfavorable responses to therapy (9).

## Receptor expression

### Somatostatin receptors

The SST family comprises 5 subtypes, SST1 through SST5. Based on sequence homology and ligand-binding characteristics, these receptors are classified into 2 subfamilies: family A (SST2, SST3, and SST5) and family B (SST1 and SST4) (48). All SSTs are G–protein-coupled receptors with 7 transmembrane domains and exert both antisecretory and antiproliferative effects (49). They are expressed in most normal tissues, with SST2 and SST5 being the most abundantly expressed subtypes. Notably, these 2 subtypes are also the predominant forms expressed in the pituitary gland, where they play a key role in regulating hormone secretion (50).

All 5 SST subtypes are expressed in somatotropinomas (49). mRNA expression analyses have shown that SST5 and SST2 are the most abundantly expressed, followed by SST3, SST1, and SST4 (51). Similar patterns are observed when the protein expression is analyzed by immunohistochemistry, with a positive correlation between the 2 techniques (52, 53).

Immunohistochemistry is the most commonly used method to assess SST expression, as it is technically simpler and can be performed on formalin-fixed, paraffin-embedded tissue, thereby eliminating the need for fresh adenoma samples (18, 52, 54). This technique uses antigen–antibody interactions to detect and localize specific proteins in tissue sections. Although relatively simple, proper immunohistochemical procedures are essential to ensure reliability and reproducibility. For instance, membrane-bound immunostaining reflects the functional status of the receptors, whereas cytoplasmic staining may be related to technical artifacts (54). Multiple antibodies—both polyclonal and monoclonal—and different scoring systems have been used for this evaluation (49). The monoclonal antibody against SST2, UMB-1, has demonstrated excellent concordance with in vitro receptor autoradiography, the gold standard method for quantifying receptor levels (55). Immunoreactivity score (IRS) is simple and reliable for evaluating SST expression (56). It is calculated by multiplying the score for the proportion of immunoreactive cells (0, 0%; 1, ≤10%; 2, 10-50%; 3, 51-80%; 4, ≥80%) by the staining intensity score (0, none; 1, weak; 2, moderate; and 3, strong), yielding a total score ranging from 0 (no expression) to 12 (highest expression) (52, 57). The IRS using the UMB monoclonal antibodies is currently considered the preferred method to quantify receptor levels (56).

We evaluated SST2 and SST5 expression in a large Brazilian multicenter study (22). High SST2 expression, defined as an IRS ≥ 6, was observed in 75% of patients and high SST5 expression was seen in 56% of patients. Biochemical control, defined as normal IGF-I (within the age-adjusted reference range) and GH < 1.0 ng/mL, was achieved in 34% of patients. Disease control was higher in patients with high SST2 expression compared with those with low expression (43% vs 8%; *P* < .001). High SST2 levels predicted biochemical control with a sensitivity of 94%, but specificity was low at 35%. The positive predictive value (PPV) was 43%, while the negative predictive value (NPV) reached 92%, resulting in an overall test accuracy of 55%. In contrast, biochemical control did not differ significantly between patients with high and low SST5 expression (22).

High SST2 expression (membranous immunostaining in at least 25% of cells) was observed in 67% of patients and was more frequent in those sensitive to octreotide or lanreotide compared with patients with resistance (persistent elevation of age-adjusted IGF-I after at least 6 months of treatment with the maximum dose of octreotide or lanreotide) (86% vs 59%; *P* = .024) (23). Patients with low SST2 expression (absence of immunostaining or immunostaining in less than 25% of tumor cells, whether cytoplasmic or membranous) had a 4.6-fold higher chance of resistance to octreotide or lanreotide (23).

A digital image analysis (DIA) system validated for quantification of SST2 expression in somatotropinomas and neuroendocrine tumors strongly correlated with the conventional IRS (Spearman's ρ: 0.969; *P* < .001) (58). SST2 expression quantified by DIA also correlated with biochemical response to octreotide or lanreotide, both when considering intensity of staining and percentage of positive cells (58). Importantly, DIA offers an objective and reproducible assessment of immunostaining, reducing interobserver variability and enabling standardized quantification across pathology laboratories.

Reports of SST expression and response to pasireotide are sparse. Pasireotide displays markedly high binding affinity for SST5, although it also binds SST2 with high affinity but lower than that of other SRLs (59). In a cohort of patients resistant to octreotide or lanreotide, biochemical response to pasireotide correlated with SST5 expression but not with SST2 or SST3 (25). No patient with low/absent SST5 expression was responsive to pasireotide (60). Nevertheless, a single study has reported that the antisecretory effect of pasireotide was mainly driven by SST2 (61).

The relation between SST expression and response to medical therapy is depicted in Table 3 (10, 19, 22, 23, 25, 27, 51, 52, 57, 58, 60-78).

**Table 3:** Correlation between somatostatin receptor subtype expression and treatment response

Author, year	N	Response to octreotide or lanreotide	Response to pasireotide
Takei, 2007 (62)	22	SST2 protein expression correlates with GH reduction in AOT	NA
Taboada, 2008 (51)	22	SST2 mRNA expression correlates positively and SST5 correlates negatively with GH and IGF-I decrease	NA
Plockinger, 2008 (63)	34	SST2 protein expression associates with biochemical response (GH)	NA
Ferone, 2008 (64)	24	SST2 protein expression correlates with IGF-I reduction	NA
Casarini, 2009 (65)	39	High SST2 protein expression associates with biochemical response (GH and IGF-I)	NA
Fougner, 2008 (66)	71	High SST2 protein expression associates with GH reduction in AOT and long-term response	NA
Gatto, 2013 (57)	36	High SST2 protein expression associates with biochemical response (IGF-I)	NA
Wildemberg, 2013 (67)	88	High SST2 protein expression associates with biochemical response (GH and IGF-I)	NA
Casar-Borota, 2013 (52)	78	High SST2 protein expression associates with biochemical response (IGF-I)	NA
Brzana, 2013 (17)	70	SST2 protein expression associates with biochemical response (GH and IGF-I)	NA
Gatto, 2016 (68)	32	SST2 protein and mRNA expression associates with biochemical response (IGF-I)	NA
Iacovazzo, 2016 (25)	39	SST2 protein expression associates with biochemical response (GH and IGF-I)	Higher SST5 protein expression associates with biochemical response (GH and IGF-I)
Venegas-Moreno, 2017 (69)	74	SST2, but not SST5, protein expression associates with biochemical response (IGF-I)	NA
Liu, 2017 (70)	93	SST2 protein expression associates with biochemical response (IGF-I) and adenoma shrinkage	NA
Coelho, 2019 (71)	96	SST2 mRNA expression associates with biochemical response (GH and IGF-I)	NA
Muhammad, 2019 (61)	52	NA	Higher SST2, but not SST5, protein expression negatively correlates with IGF-I after treatment
Coopmans, 2020 (72)	45	NA	Lower SST2 expression associates with adenoma shrinkage. No association with SST5
Wildemberg, 2021 (10)	101	SST2 protein and mRNA expression associates with biochemical response (GH and IGF-I)	NA
Wildemberg, 2021 (22)	153	SST2 protein expression associates with biochemical response (GH and IGF-I)	NA
Berton, 2022 (23)	96	Low SST2 protein expression more frequent in patients with resistance (IGF-I)	NA
Chiloiro, 2021 (60)	74	NA	Low SST5 protein expression associates with adverse GH and IGF-I response
Campana, 2022 (58)	39	SST2 protein expression associates with biochemical response (IGF-I)	NA
Ilie, 2022 (73)	49	SST2, but not SST5, protein expression correlates with biochemical response (IGF-I)	No correlation with SST2 or SST5 protein expression
Gligla, 2024 (27)	34	SST2 protein expression correlates with biochemical response (IGF-I)	SST5 protein expression associates with biochemical response (IGF-I)
Luo, 2024 (74)	35	SST2 protein expression associates with biochemical response (IGF-I)	NA

Abbreviations: AOT, acute octreotide test; GH, growth hormone; IGF-I, insulin-like growth factor 1; NA, not available; SST2, somatostatin receptor subtype 2; SST5, somatostatin receptor subtype 5.

### Dopamine receptors

Dopamine receptors consist of 5 G–protein-coupled receptor subtypes (79) classified as D1-like receptors, which include D1R and D5R, and D2-like receptors, comprising D2R, D3R, and D4R (29). These receptors are abundantly expressed in the central nervous system, pituitary gland, kidneys, and vasculature (79). D2R is the most expressed subtype in the normal pituitary gland and in somatotropinomas (80). D2R expression has limited value in predicting response to cabergoline and does not determine SRL responsiveness (69, 80).

## Translating pathological and receptor expression features into clinical management

Histological subtypes and receptor expression profiles can determine both surgical and medical therapy outcomes. These include variations in adenoma granulation pattern, PIT-1 lineage differentiation, and SST expression. The challenge lies in how these pathological and receptor expression features can be effectively validated and integrated into the treatment decision-making process.

A study evaluating clinical, biochemical, imaging, and immunohistochemical features in a cohort of 292 patients identified 3 distinct patient subgroups, each associated with specific adenoma biology, receptor expression profiles, and treatment outcomes (75). The first group (Type 1) comprised older individuals with less aggressive, densely granulated adenomas exhibiting high SST2 expression, lower GH and IGF-I levels, and a favorable response to medical therapy. The second group (Type 3) included younger patients with more aggressive and typically sparsely granulated adenomas characterized by low SST2 expression and poor responsiveness to treatment. The third intermediate group (Type 2) included patients with both densely and sparsely granulated adenomas with nonaggressive behavior, moderate SST2 expression, and a variable response to therapy (75). This integrative classification underscores how combining histopathological and receptor-based parameters can refine disease stratification and guide a more personalized therapeutic approach in acromegaly.

Interestingly, signal intensity on T2-weighted MRI sequences has been associated with both granulation pattern and SST2 expression and response to therapy (4). Hypointense adenomas are more frequently densely granulated and exhibit a more favorable biochemical parameter and adenoma volume reduction in response to octreotide or lanreotide (81, 82). It could be inferred that patients with hypointense adenomas are more often classified as Type 1, while those with iso-/hyperintense adenomas correspond more frequently to Type 3. This imaging characteristic may serve as a preoperative marker of both surgical and medical treatment.

In the Brazilian multicenter study, machine learning techniques were applied to integrate age at diagnosis, sex, pretreatment GH and IGF-I levels, SST2 and SST5 expression, and granulation pattern as biomarkers underlying a prediction model for biochemical control with octreotide and lanreotide (22). This model achieved an accuracy of 86.3% for predicting biochemical control, substantially higher than that of SST2 expression alone (55%) (22). Other prediction models have also been developed (83-86).

A French study prospectively evaluated 47 patients treated postoperatively with octreotide (73). Densely granulated adenomas exhibited higher SST2 expression and more suppressed GH and IGF-I levels after treatment. In contrast, sparsely granulated adenomas showed higher SST5 expression (73).

When therapeutic decisions were prospectively made according to established clinical guidelines as compared with a biomarker-guided strategy, patients were assigned to receive octreotide or lanreotide, pegvisomant, or a combination of both, based on SST2 expression, T2-weighted MRI signal intensity, granulation pattern, and baseline IGF-I levels (87). In the personalized treatment group, 78% of patients achieved normal IGF-I levels, compared with 53% in the standard treatment group (*P* = .04). The personalized approach was associated with a 2.53-fold higher likelihood of achieving biochemical control (95% CI: 1.30-4.80). Moreover, patients in the personalized group achieved biochemical control approximately 4 months earlier than those in the standard group (median of 182 vs 305 days; *P* = .06) (87).

In the most recent consensus on therapeutic outcomes, assessment of granulation pattern was recognized as the minimum essential criterion for predicting SRL responsiveness (88). SST2 immunopositivity may also predict treatment response. The consensus also highlighted the potential value of models integrating multiple biomarkers to enhance the accuracy of treatment response prediction. Importantly, the treatment algorithm incorporated predictive factors associated with a favorable response to therapy, including hypointense T2-weighted MRI signal, high SST2 expression, densely granulated adenoma subtype, and lower baseline GH and IGF-I levels. These features support the use of octreotide or lanreotide as initial therapy. Conversely, in patients with adverse biomarkers indicating poor response, pegvisomant could be considered. When biochemical control is not achieved with octreotide or lanreotide, high SST5 expression may be a reason to switch to pasireotide (88), representing an important step toward a biomarker-driven, personalized treatment strategy for acromegaly.

**Figure 1:**
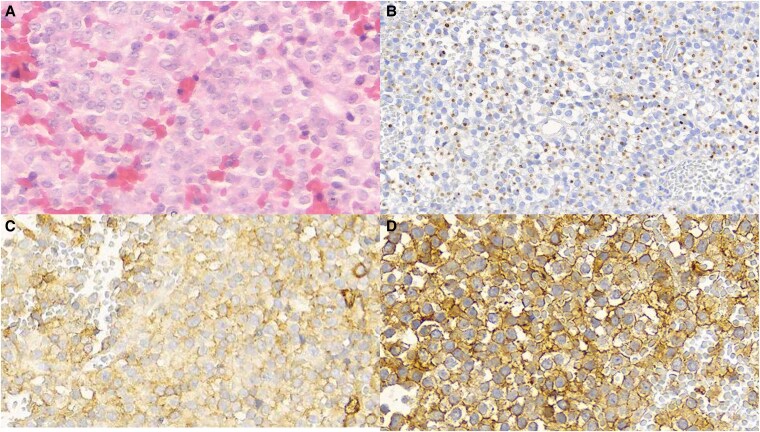
Histopathological and immunohistochemical features. **(**A**)** Hematoxylin–eosin staining showing a sparsely granulated pituitary adenoma. **(**B**)** CAM5.2 immunostaining demonstrating the characteristic perinuclear dot-like cytokeratin pattern. **(**C**)** Somatostatin receptor subtype 2 (SST2) immunohistochemistry showing mainly cytoplasmic staining (low expression). **(**D**)** Somatostatin receptor subtype 5 (SST5) immunohistochemistry demonstrating strong and diffuse expression (high expression).

To illustrate the clinical relevance of biomarkers of response, we briefly describe a young female patient with classical clinical acromegaly, including markedly elevated GH levels, moderately elevated IGF-I levels, and a large invasive macroadenoma. The patient underwent transsphenoidal surgery, and histopathological/immunohistochemical analysis revealed a sparsely granulated somatotroph adenoma with low SST2 expression (IRS 2) and high SST5 expression (IRS 12) (Fig. 1). Because of persistent disease, the patient was subsequently treated with lanreotide Autogel 120 mg every 4 weeks, without achieving biochemical control or adenoma shrinkage. Pretreatment GH and IGF-I levels (63 ng/mL and 2.46× upper limit of normal [ULN], respectively) were unchanged after 6 months of treatment. Treatment was altered to pasireotide 40 mg, with IGF-I normalization after 3 months (0.83× ULN).

Implementation of personalized management still faces challenges because, although granulation pattern and SST2 expression are key predictors, other biomarkers may also provide meaningful value. The optimal combination of biomarkers for guiding treatment decisions, nonetheless, remains to be established. Moreover, heterogeneity in methodologies and quantification strategies across studies complicates data interpretation, underscoring the need for standardization before these biomarkers can be reliably applied in clinical practice. Another important limitation is the uneven global availability of advanced biomarker testing.

Notably, some may argue that acromegaly, being a chronic and slowly progressive disease, is unlikely to be significantly affected by a short delay in achieving disease control. In contrast, initiating treatment with more potent drugs that carry a higher risk of adverse effects may expose certain patients to unnecessary risks. Therefore, additional prospective trials comparing a standard stepwise therapeutic approach with biomarker-guided strategies are warranted to assess the long-term clinical and safety of personalized treatment algorithms. Identification of biomarkers associated with poor treatment response should prompt early dose uptitration, consideration of combination therapy, or timely switching to an alternative medical approach.

## Conclusion

Incorporating granulation pattern and SST2 expression as biomarkers into evidence-based decision-making has improved the prediction of treatment response, particularly to SRLs, although the correlation between granulation pattern and surgical remission remains debatable. Proper histological classification of adenomas may also be valuable in predicting treatment response. While current evidence supports a more personalized, biomarker-guided approach, additional prospective studies are needed to confirm its long-term superiority over conventional stepwise treatment strategies and facilitate broader clinical integration.

## Data Availability

Data sharing is not applicable to this article as no data sets were generated or analyzed during the present study.

## Abbreviations

CI: confidence interval
CK8/18: cytokeratin 8/18
DIA: digital image analysis
IGF-I: insulin-like growth factor 1
IRS: immunoreactivity score
MRI: magnetic resonance imaging
NPV: negative predictive value
OR: odds ratio
PPV: positive predictive value
PRL: prolactin
SRL: somatostatin receptor ligand
SST: somatostatin receptor
SST1: somatostatin receptor subtype 1
SST2: somatostatin receptor subtype 2
SST3: somatostatin receptor subtype 3
SST4: somatostatin receptor subtype 4
SST5: somatostatin receptor subtype 5
T2: transverse relaxation time
TSH: thyroid-stimulating hormone
ULN: upper limit of normal
WHO: World Health Organization
